# Maternal and protective antibody responses to E2 subunit and C-strain classical swine fever vaccines in piglets: a comparative analysis

**DOI:** 10.3389/fvets.2025.1694586

**Published:** 2025-11-10

**Authors:** Hongwei Ma, Mengen Xu, Jie Yang, Xiaohu Zhang, Bo Liu, Zuohua Li, Zhen Liu, Wei Hu, Wei Fan, Jiakui Li

**Affiliations:** 1 College of Veterinary Medicine, Huazhong Agricultural University, Wuhan, China; 2 Pulike Bioengineering Co., Ltd., Luoyang, China; 3 Hubei Jiangxia Laboratory, Wuhan, China; 4 Rongai Breeding Specialized Professional Cooperative, Macheng, China

**Keywords:** CSFV, C-strain vaccine, E2 subunit vaccine, immunization, protective efficacy

## Abstract

Classical swine fever (CSF), caused by the classical swine fever virus (CSFV), is an acute and highly contagious disease affecting swine. In clinical settings, the C- strain vaccine and the E2 subunit vaccine are commonly employed for the prevention and management of the condition. This study monitored the antibody levels of pigs immunized with different CSF vaccines under field conditions, comparing the effects of the classical swine fever virus E2 subunit vaccine and the C-strain vaccine on the duration of maternal antibody maintenance and protective efficacy in fattening pig herds. In the case of sows vaccinated with the E2 subunit vaccine, effective maternal antibodies can be maintained for up to 75 days postpartum, thereby allowing the first vaccination of their piglets to be delayed until 75 days of age. With regard to the immunization of fattening pigs, the E2 subunit vaccine has been demonstrated to confer protection for a period of time sufficient to allow the animals to reach market weight following a single administration of the vaccine. The findings demonstrate that, in comparison with the C-strain vaccine, the E2 subunit vaccine has the capacity to sustain maternal antibodies and protective antibodies over a more protracted period without the necessity for booster immunizations. This translates to a reduction in vaccination frequency and dosage requirements in commercial production. This study provides a theoretical foundation for the optimization of immunization processes within clinical practice.

## 1 Introduction

Classical swine fever (CSF) is a highly contagious viral disease affecting pigs. In addition to domestic pigs, wild boars and feral pigs are also exposed to the threat of the disease, causing significant damage to the global pig farming industry (1). The causative agent, Classical swine fever virus (CSFV), belongs to the *Flaviviridae* family and the *Pestivirus* genus (2). CSFV is an enveloped virus with a single-stranded, positive-sense RNA genome and an icosahedral capsid (3). The approximately 12.3 kb genome encodes a polyprotein of about 3,898 amino acids, which is processed into 12 mature proteins: four structural proteins (core protein (C), envelope glycoproteins E^rns^, E1, and E2) and eight non-structural proteins (N^pro^, p7, NS2, NS3, NS4A, NS4B, NS5A, and NS5B) (4–6). In addition, the categorization of CSFV genotypes is predominantly delineated into three primary types (1, 2, and 3). Within these categories, genotype 1 is further subdivided into seven subtypes (1.1–1.7), genotype 2 is subdivided into seven subtypes (2.1–2.7), and genotype 3 is subdivided into seven subtypes (3.1–3.4) (7).

The earliest documented reports of classical swine fever, date back to 1810 in the US state of Tennessee. Following the expansion of the US railway system after 1860, the disease spread rapidly across the country (8). CSF first manifested in the UK in Europe in 1862, subsequently spreading to Sweden, France and Denmark. By the 1960s, the disease had spread globally (9). The initial outbreak of CSF in China was not meticulously documented. Chinese researchers first trialed CSF therapeutic hyperimmune serum in 1925, and the first highly pathogenic CSFV strain in China was isolated in 1945 (10). The distribution of CSFV genotypes exhibits distinct regional characteristics. Genotype 3 is found exclusively in Asia, while field isolates in the Americas are all genotype 1, and viral isolates in Europe are all genotype 2. On a global scale, genotype 2 has been the most prevalent genotype over the past few decades (7, 8). Presently, a multitude of genotypes of the virus have been identified in mainland China, including 2.1, 2.2, 2.3, and 1.1, with the 2.1 subtype having been the dominant strain in China for an extended period (11, 12).

The presence of CSF has resulted in substantial economic losses for countries with extensive pig production. In an effort to address this challenge, two primary strategies have been employed: systematic vaccination and non-vaccination culling (13, 14). It is evident that, in view of the considerable expense associated with culling, systematic preventive vaccination constitutes a more efficacious strategy for the control of CSF in countries where it is endemic (15). Early CSF vaccines were developed in the early 20th century. By the 1940s, modified live vaccine (MLV) had been developed through the process of passaging highly virulent strains through rabbits (16). Among these, the Chinese vaccine strain (C-strain), also known as the “Chinese hog cholera lapinized virus” (HCLV), was extensively utilized in numerous countries due to its high safety and remarkable efficacy (17). However, as serological differentiation between infected and vaccinated animals (DIVA) is impossible with MLV, an E2 subunit marker vaccine was developed (18, 19). Despite the evident success of the CSF virus vaccine in pig populations, there is a paucity of research on the duration of maternal antibodies and protective antibodies induced by the E2 subunit vaccine, and its impact on the production performance of fattening pigs. This study monitored the persistence of maternal antibodies and protective antibodies following administration of the immune E2 subunit vaccine and C-strain vaccine in clinical production settings. Furthermore, the study made a comparative analysis of the effects of different vaccines on pig production performance, providing valuable insights for farms selecting CSF vaccines and optimizing production efficiency.

## 2 Materials and methods

### 2.1 Vaccines and animals

The CSF E2 subunit vaccine (batch no.: 552212002) and C-strain vaccine (batch no.: 122212077) utilized in this study were provided by Pulike Biological Engineering, Inc. The immunological trial was conducted at a large-scale commercial pig farm in Huanggang City, Hubei Province, China. The experimental animals comprised crossbred sows (2–3 parity) one month prior to farrowing and weaning of piglets.

### 2.2 Experimental design

A total of 182 healthy sows in the pig farm, one month before farrowing, were randomly divided into two groups: Group A (142 sows) and Group B (40 sows). Group A was immunized with the E2 subunit vaccine, while Group B was immunized with the C-strain vaccine. The 1,807 piglets in the pig farm that were about to be weaned were randomly divided into two groups: Group C (1,000 piglets) and Group D (807 piglets). Group C was immunized with the E2 subunit vaccine, while Group D was immunized with the C-strain vaccine (20). The dosage and number of immunizations were administered in accordance with the instructions outlined in the manual, with specific details listed in Table 1. During the trial period, the pigs were fed and managed in accordance with the pig farm’s daily management procedures.

**Table 1:** Immunization dosage and timing.

Time	A	B	C	D
30 days pre-farrowing	E2, 1 dose	C-strain, 1 dose		
25-day-old		C-strain, 1 dose (piglets produced)	E2, 1 dose	C-strain, 1 dose
55-day-old		C-strain, 1 dose (piglets produced)		C-strain, 1 dose

### 2.3 Sample collection and indicator analysis

The feeding behavior, body temperature, and clinical sign of each group were monitored and recorded. For groups A and B, the number of piglets born, average piglet birth weight, and mortality during the experimental period are to be recorded. Blood samples were collected on the day of birth (umbilical cord blood), at 16, 25, 45, and 75 days of age. Following the process of allowing the blood to settle and centrifuging to separate the serum, the antibody levels for CSFV were detected. Serum samples were collected from Groups C and D at 16, 35(2 weeks post-vaccination), 50 (4 weeks post-vaccination), 80 (8 weeks post-vaccination), 123 (14 weeks post-vaccination), 162 (20 weeks post-vaccination) and 200 days of age (25 weeks post-vaccination). The serum samples collected by each group were randomly selected and comprised 15 specimens each. Serum CSFV antibody levels were determined using the Classical Swine Fever Virus Blocking ELISA Antibody Detection Kit (Putai, Luoyang, China) in accordance with the manufacturer’s instructions. The final result is calculated based on the OD value measured at 450 nm wavelength using the formula provided in the kit instructions.

### 2.4 Data analysis

Statistical analysis and graphical representation of the data were conducted using GraphPad Prism 8.0 software. The data are expressed as the mean ± standard deviation. Intergroup comparisons were performed using the one-sample t-test. Statistically speaking, a *p* value less than 0.05 was deemed to be significant.

## 3 Result

### 3.1 Vaccine safety and impact on sow reproductive performance

Following immunization, pigs in all groups exhibited normal feeding and mental status, with body temperatures remaining within the normal range. No instances of immune stress or other adverse effects were observed. The average number of healthy piglets per litter for sows in Groups A and B was 13.12 and 12.97, respectively, with an average birth weight of 1.31 kg and 1.33 kg per piglet, respectively. By 75 days of age, the mortality rates for piglets born to sows in the two groups were 6.23 and 6.17%, respectively, with no significant difference between the two groups. Detailed data is presented in Table 2.

**Table 2:** The effects of two vaccines on the reproductive performance of sows.

Parameter	A	B
Number of immune samples	142	40
Total number of healthy piglets	1863	519
Average number of healthy piglets per litter	13.12	12.97
Average birth weight of healthy piglets (kg)	1.31	1.33
Number of healthy piglets at 75 days of age	1747	487
Mortality rate from birth to 75 days of age (%)	6.23	6.17

### 3.2 E2 subunit vaccine provides an extended duration of maternal antibodies than C-strain vaccine

**Figure 1:**
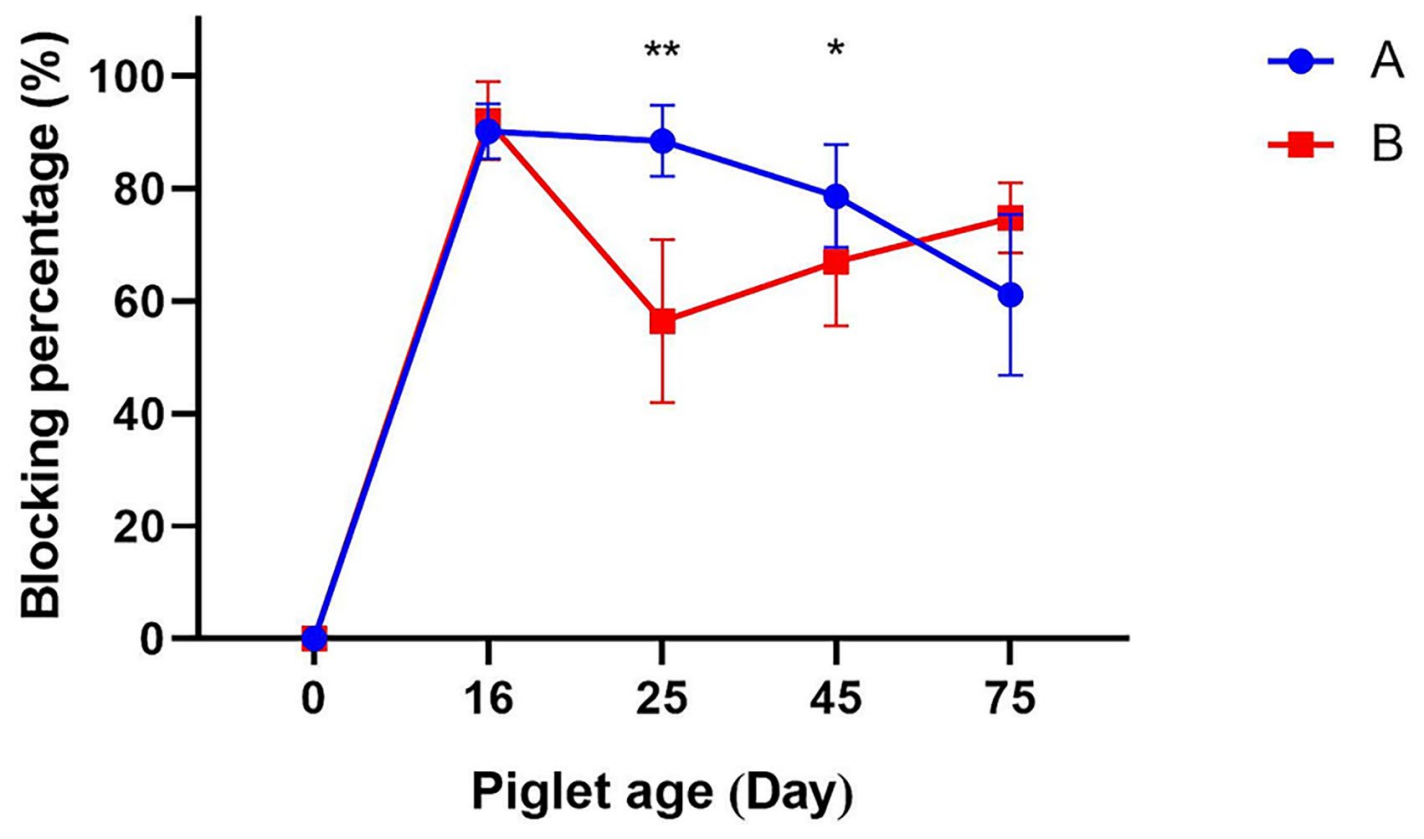
The effect of two vaccines on maternal antibodies. A: Group A was immunized with the E2 subunit vaccine. B: Group B was immunized with the C-strain vaccine. * *p* < 0.05 and ** *p* < 0.01.

The level of maternal antibodies in sows after immunization is reflected by the CSFV antibody blocking percentage of their piglets. The antibody levels of CSFV in piglets produced by Group A decreased with increasing age, but remained above 70% until 75 days of age. The CSFV antibody levels in piglets born in Group B were above 90% at 16 days of age, but then fell quickly, dropping below 70% by 25 days of age and remaining significantly lower than those in Group A. In order to ensure the provision of immune protection for the batch of piglets in question, the primary immunization was administered in the form of the C-strain vaccine on the 25th day of age. Subsequent to the primary vaccination, an increase in antibody levels was observed in Group B piglets. At 45 days of age, the antibody levels increased to 66.93 ± 11.93%, remaining at a low level that did not meet the threshold for herd immunity and was significantly lower than that of Group A (78.63 ± 9.12%). Following the administration of a booster vaccination at 55 days of age, an increase in antibody levels was observed, reaching 74.80 ± 6.3% at 75 days of age (Figure 1).

### 3.3 E2 subunit vaccine protects fattening pigs for longer than the C-strain vaccine

**Figure 2:**
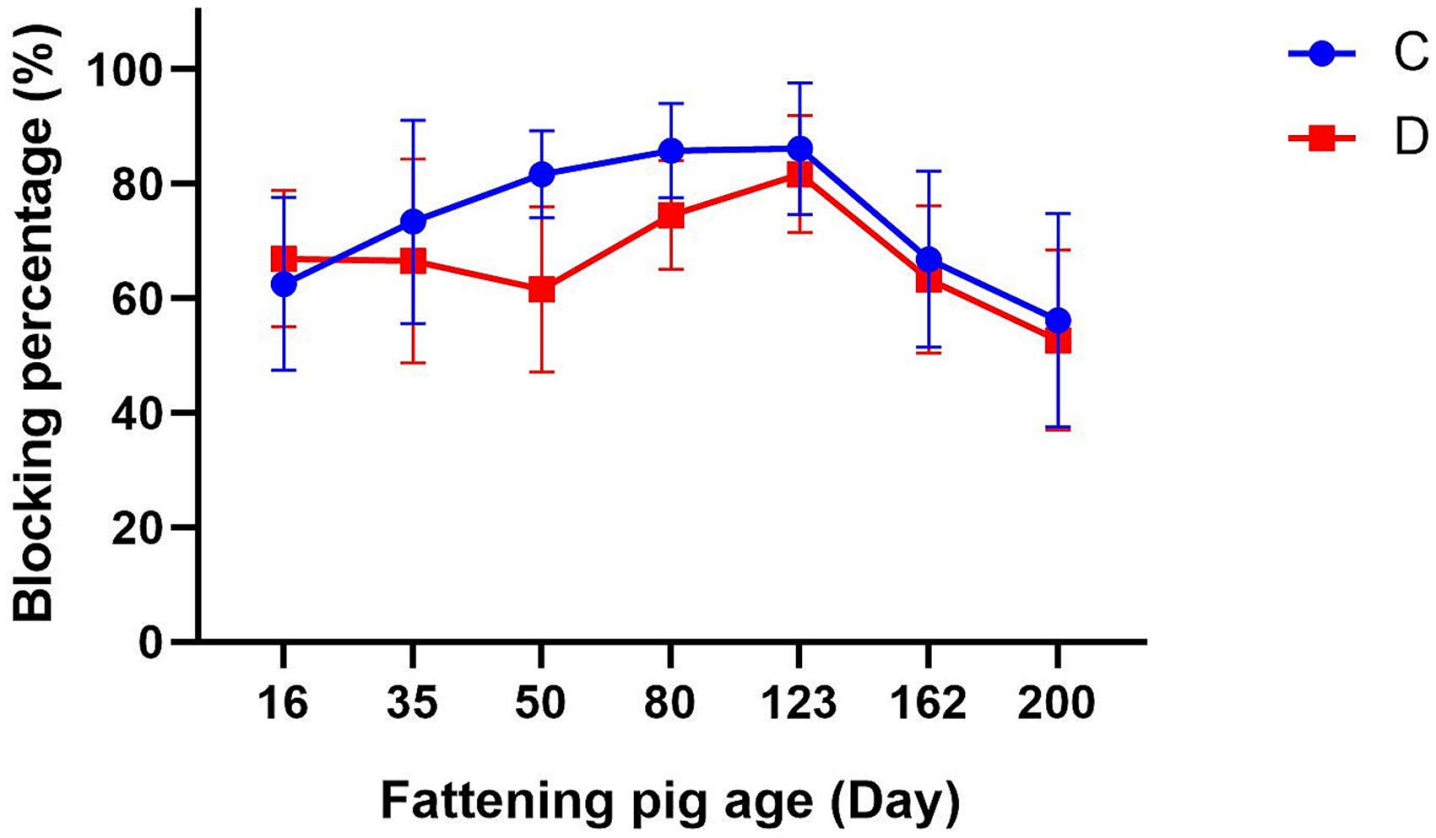
The effect of two vaccines on protective antibodies. C: Group C was immunized with the E2 subunit vaccine. D: Group D was immunized with the C-strain vaccine.

The CSFV antibody levels of fattening piglets in groups C and D were both higher than the specified standard before immunization, and no significant difference was observed between the two groups. Subsequent to immunization, the CSFV antibody levels in Group C demonstrated a sustained upward trajectory until 14 weeks post-immunization. Thereafter, the antibody levels initiated a decline, yet persisted at approximately 60% at 25 weeks post-immunization. Group D did not demonstrate a significant increase in antibody levels during the initial two weeks following immunization. By week 4, a decline in antibody levels to 61.54 ± 14.38% was observed. Subsequent to booster vaccination of the swine in Group D at 55 days of age, an increase in antibody levels was observed, albeit with a subsequent decline commencing the 14th week post-primary vaccination (Figure 2).

## 4 Discussion

CSFV has had a significant impact on China’s pig farming industry, resulting in substantial economic losses. In response, the Chinese government has implemented a range of measures to address the issue, including the mandatory administration of live C-strain vaccines, which are widely regarded as both safe and effective (21). In light of the ongoing advancements in the field of vaccine development, the E2 subunit vaccine has been authorized for utilization, thereby providing farmers with a more extensive range of options, albeit concomitantly complicating the decision-making process (22). In this experiment, no adverse reactions were observed in any of the four groups of pigs (A, B, C, and D) following vaccination. Furthermore, no significant disparities were observed in litter size, average birth weight, or mortality rate at 75 days of age between the A and B groups of sows. This finding indicates that the E2 subunit vaccine demonstrates a comparable level of safety to the C-strain vaccine under field conditions.

Following vaccination, an increase in antibody levels is observed, yet the immune effect concomitantly diminishes over time. Consequently, the judicious selection of the optimal time for booster vaccinations can ensure the provision of prolonged immune protection (23). Suckling piglets are provided with protection against CSFV infection primarily through maternal antibodies during the early stages of life (24). In current porcine production systems, breeding sows are vaccinated with the C-strain vaccine four times per year. Piglets are typically primed at 25–35 days of age and receive a booster one month later. Nevertheless, there are still frequent cases of inadequate antibody levels. Our research findings indicate that maternal antibodies produced by sows vaccinated with the C-type vaccine 30 days prior to farrowing persist only until 25 days after piglet birth, at which point they fall below the 70% standard commonly used in actual production. It is therefore necessary to administer the primary vaccination during this period, which is consistent with standard field protocols. Furthermore, in fattening pigs, antibody levels remained stable without a significant increase after the first C-strain vaccination, likely due to interference from maternal antibodies. However, a clear rise in antibody titers was observed following booster vaccination, confirming the necessity of boosters and supporting the rationale underlying the current immunization program.

As the principal target for immune responses, the E2 protein of CSFV is responsible for inducing potent and durable neutralizing antibody titers (13, 25, 26). This process serves to prevent viral invasion and thus provide protection against CSFV in pig populations. This study demonstrated that the E2 subunit vaccine sustained maternal antibody persistence significantly longer than the C-strain vaccine, maintaining titers above the protective threshold until piglets reached 75 days of age. These results indicate that maternal antibodies alone can confer protection against CSFV infection without active immunization, allowing for a delayed primary vaccination schedule in piglets until 75 days of age. Gong’s research confirmed that the E2 subunit vaccine can protect against four strains of classical swine fever type 2. Furthermore, it was established that the protective effect of the E2 subunit vaccine is equivalent to that of the traditional C-strain live vaccine (26). Terzić S also ascertained that the E2 subunit vaccine can elicit antibodies more rapidly and efficiently (27). Consistent with these findings, a single dose of the E2 vaccine induced a sustained increase in antibody titers that remained elevated until 123 days of age. At market age (200 days), pigs in Group C maintained antibody levels of approximately 60%, higher than those in Group D, which received a booster vaccination. These results demonstrate that under field conditions, a single E2 subunit vaccination administered at 75 days of age is sufficient to provide protective immunity until market readiness.

## 5 Conclusion

In summary, this study evaluated antibody dynamics induced by different CSFV vaccines under field production conditions. We confirm that maternal protective antibodies derived from the C strain live vaccine decline below the regulatory threshold commonly used in actual production by 25 days of age, whereas antibodies derived from the E2 subunit vaccine extend this period to 45–75 days of age. Moreover, a single dose of the E2 vaccine provided prolonged immunity in fattening pigs until market age, while the C-strain vaccine required booster immunizations to maintain protection. As this investigation was conducted under field conditions, the findings offer practical value for commercial pig operations seeking to reduce the frequency and complexity of classical swine fever vaccination protocols.

## Data Availability

The raw data supporting the conclusions of this article will be made available by the authors, without undue reservation.
